# A Food Intake Estimation System Using an Artificial Intelligence–Based Model for Estimating Leftover Hospital Liquid Food in Clinical Environments: Development and Validation Study

**DOI:** 10.2196/55218

**Published:** 2024-11-05

**Authors:** Masato Tagi, Yasuhiro Hamada, Xiao Shan, Kazumi Ozaki, Masanori Kubota, Sosuke Amano, Hiroshi Sakaue, Yoshiko Suzuki, Takeshi Konishi, Jun Hirose

**Affiliations:** 1 Medical Informatics, Institute of Biomedical Sciences Tokushima University Graduate School Tokushima Japan; 2 Department of Therapeutic Nutrition, Institute of Biomedical Sciences Tokushima University Graduate School Tokushima Japan; 3 Medical Information Technology Center Tokushima University Hospital Tokushima Japan; 4 Department of Oral Health Care Promotion, Institute of Biomedical Sciences Tokushima University Graduate School Tokushima Japan; 5 foo.log Inc Tokyo Japan; 6 Division of Nutrition Tokushima University Hospital Tokushima Japan; 7 Department of Nutrition and Metabolism, Institute of Biomedical Sciences Tokushima University Graduate School Tokushima Japan

**Keywords:** artificial intelligence, machine learning, system development, food intake, dietary intake, dietary assessment, food consumption, image visual estimation, AI estimation, direct visual estimation

## Abstract

**Background:**

Medical staff often conduct assessments, such as food intake and nutrient sufficiency ratios, to accurately evaluate patients’ food consumption. However, visual estimations to measure food intake are difficult to perform with numerous patients. Hence, the clinical environment requires a simple and accurate method to measure dietary intake.

**Objective:**

This study aims to develop a food intake estimation system through an artificial intelligence (AI) model to estimate leftover food. The accuracy of the AI’s estimation was compared with that of visual estimation for liquid foods served to hospitalized patients.

**Methods:**

The estimations were evaluated by a dietitian who looked at the food photo (image visual estimation) and visual measurement evaluation was carried out by a nurse who looked directly at the food (direct visual estimation) based on actual measurements. In total, 300 dishes of liquid food (100 dishes of thin rice gruel, 100 of vegetable soup, 31 of fermented milk, and 18, 12, 13, and 26 of peach, grape, orange, and mixed juices, respectively) were used. The root-mean-square error (RMSE) and coefficient of determination (*R*^2^) were used as metrics to determine the accuracy of the evaluation process. Corresponding *t* tests and Spearman rank correlation coefficients were used to verify the accuracy of the measurements by each estimation method with the weighing method.

**Results:**

The RMSE obtained by the AI estimation approach was 8.12 for energy. This tended to be smaller and larger than that obtained by the image visual estimation approach (8.49) and direct visual estimation approach (4.34), respectively. In addition, the *R*^2^ value for the AI estimation tended to be larger and smaller than the image and direct visual estimations, respectively. There was no difference between the AI estimation (mean 71.7, SD 23.9 kcal, *P*=.82) and actual values with the weighing method. However, the mean nutrient intake from the image visual estimation (mean 75.5, SD 23.2 kcal, *P*<.001) and direct visual estimation (mean 73.1, SD 26.4 kcal, *P*=.007) were significantly different from the actual values. Spearman rank correlation coefficients were high for energy (ρ=0.89-0.97), protein (ρ=0.94-0.97), fat (ρ=0.91-0.94), and carbohydrate (ρ=0.89-0.97).

**Conclusions:**

The measurement from the food intake estimation system by an AI-based model to estimate leftover liquid food intake in patients showed a high correlation with the actual values with the weighing method. Furthermore, it also showed a higher accuracy than the image visual estimation. The errors of the AI estimation method were within the acceptable range of the weighing method, which indicated that the AI-based food intake estimation system could be applied in clinical environments. However, its lower accuracy than that of direct visual estimation was still an issue.

## Introduction

### Background

Inadequate diet and nutritional intake are causes of malnutrition [1]. Food intake is the primary criterion for assessing malnutrition among patients [2]. Medical staff often conduct surveys, such as food intake and nutrient sufficiency ratios, to accurately assess patients’ food intake. Typical methods for measuring food intake include weighing and visual estimation. Although weighing foods before and after consumption is the most accurate method, it is burdensome for the measurer [3]. In the visual estimation method, nurses, caregivers, and other medical staff estimate and record food intake through direct observation. However, this is difficult with numerous patients. Visual estimation is popular as it is significantly associated with the weighing method and is a valid assessment [4]. However, only some patients measure their food intake [5]. Nursing staff failed to record 44% (220/503) of meals correctly when they used a food intake chart to record all the meals consumed by patients [6]. Therefore, other methods may be useful for recording food intake in hospitals, such as confirming and recording the amount of food intake as determined by the patient’s visual estimation [7]. However, this method does not provide an accurate measurement as the visual estimation method may not be precise if performed without training [8,9]. These factors make food intake measurement both complicated and inaccurate in some clinical environments.

As a solution, various mobile apps have been developed to record food intake and manage calorie and nutrient intake from meals [10]. In particular, systems that managed daily food intake and recommended personalized meals and recipes were useful for weight loss [11]. However, nutrition management applications that require manually inputting meal contents to calculate calorie and nutrient intake demonstrated accuracy issues, such as varying intakes between applications [12,13] and significant underestimation of energy intake [14]. Therefore, these systems should be further improved for use in clinical settings. In addition, some applications introduced artificial intelligence (AI), such as estimating calories from food images [15]. However, AI cannot accurately manage the amount of food a patient actually consumes as it shows the result of the entire intake. Therefore, we developed an AI model to estimate leftover liquid food in hospitals through a convolutional neural network and aimed to achieve further accurate measurements. The mean absolute error (MAE) from this AI estimation was 0.85, which was significantly smaller than 1.03 (*P*=.009) in the visual estimation of the food images by the dietitians. This indicates an error of 8.5% with the weighing method. The high accuracy of this AI model was indicated as measurements in clinical settings should have an error margin of less than 10% when using the weighing method [16]. However, this evaluation was based on images of hospital liquid food captured by a camera.

### Objective

We evaluated the accuracy of the AI estimates for liquid foods actually served to hospitalized patients. We built a system that could manage the food intake of multiple patients admitted to a hospital through our AI model to estimate leftover food. A system that can automatically and accurately determine patients’ food intake by photographing and uploading liquid food after a meal through mobile devices can increase accessibility for medical staff.

## Methods

### Construction of a Food Intake Estimation System by an AI Model to Estimate Leftover Food

**Figure 1:**
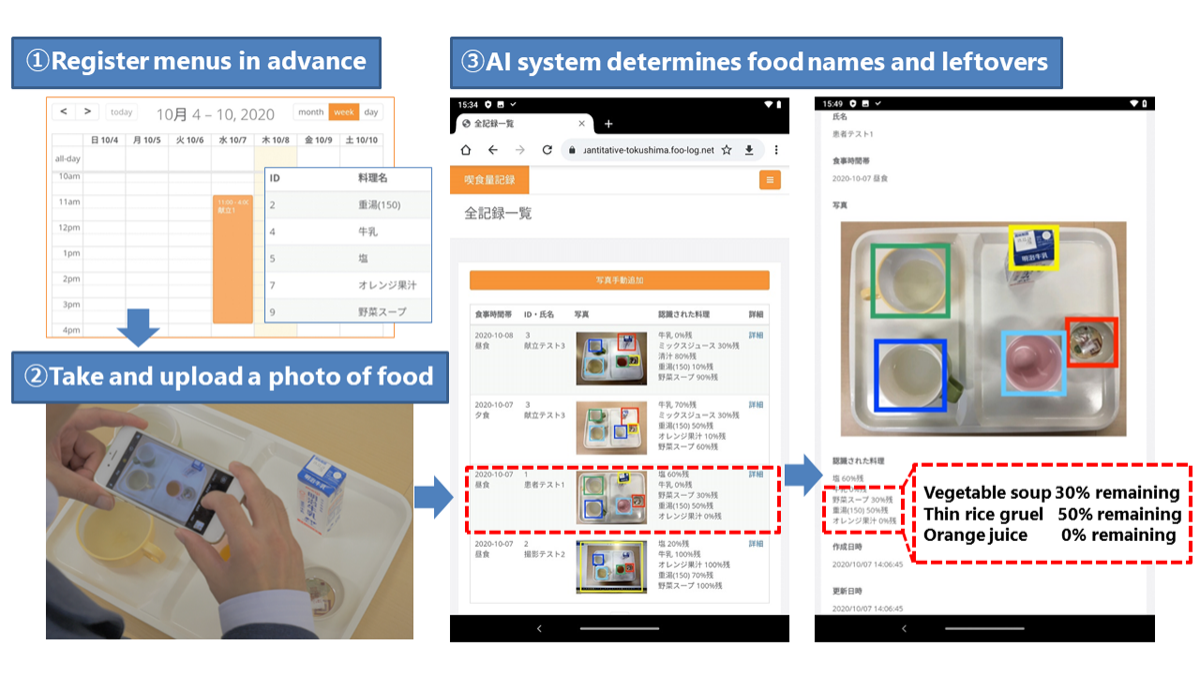
Overview of food intake estimation system.

**Figure 2:**
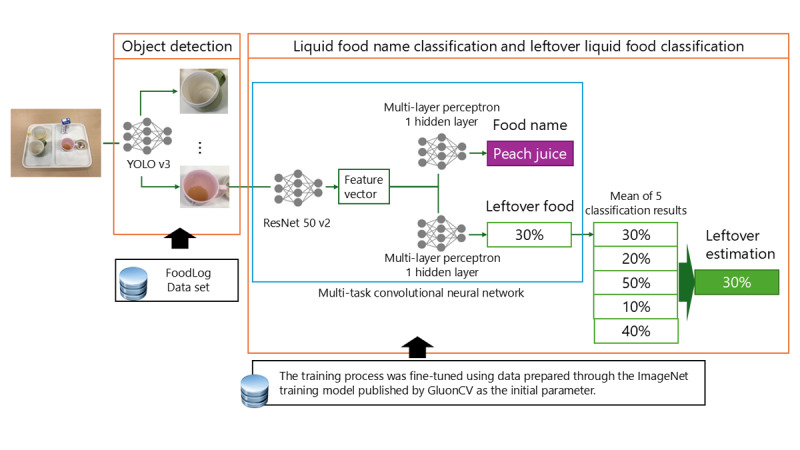
Artificial intelligence model to estimate leftover liquid food.

The food intake estimation system (Figure 1) matched the photographed foods with a menu preregistered by the administrator. Menu information is obtained from the dietitian and registered in this system. Users took a picture of the food after a meal through their mobile devices and uploaded the image to the food intake estimation system server. The system is designed to upload images according to each individual’s meal menu. The AI analyzed the data from the uploaded image to determine the area, name, and leftover food. In addition, the determined contents were displayed on the screen. We used our previously developed AI model to estimate leftover food [16]. The AI model comprises 2 parts: an object-detection part that identifies the positions of multiple dishes on a tray and extracts their regions from a single liquid food image and a leftover-estimation part that classifies the names of liquid foods associated with the detected objects and estimates the amount of leftover liquid food (Figure 2). The leftover estimation is a task that comprises classifying leftover liquid food on an 11-point scale (0%, 10%, 20%, 30%, 40%, 50%, 60%, 70%, 80%, 90%, and 100%). A convolutional neural network analyzed the liquid food images. Data transmission from mobile devices to the server was through the internet. Furthermore, the encryption used transport-layer security. The system is encrypted and authenticated using TLS1.2 ECDHE_RSA with P-256 and AES_128_GCM.

### Dataset for Training

We trained the AI using our previously developed dataset, with additional training on images where 5% or less of the entire amount was ingested [16]. The liquid food menu consisted of a staple food, 2 side dishes, packaged beverage, and seasonings. Table 1 lists the types of dishes and a number of images used for AI training. The images consisted of a combination of portions created to include 0%, 10%, 20%, 30%, 40%, 50%, 60%, 70%, 80%, 90%, and a state with no leftovers of each liquid food. An annotation tool (visual object tagging tool) was used to label the area, name, and leftover food on the tray. Furthermore, the image was divided into separate pictures for each dish. Liquid food images were taken under various lighting conditions for breakfast, lunch, and dinner on multiple dates and times.

**Table 1:** Types of dishes and number of images used for artificial intelligence training.

Type of food and liquid food name		Training images, n
Staple food: thin rice gruel		576
**Side dishes 1**		
	Japanese clear soup	144
	Vegetable soup	432
	Miso soup	144
	Red miso soup	66
**Side dishes 2**		
	Fermented milk	84
	Peach juice	84
	Grape juice	84
	Orange juice	84
	Mixed juice	78
	Fruit mix	78
**Packaged beverage**		
	Milk	576
	Milk for toddlers	66
	Apple juice for toddlers	66
	Orange juice for toddlers	66
	Additive-free vegetable juice	66
Seasoning: salt		576

### Dataset for Evaluation

**Figure 3:**
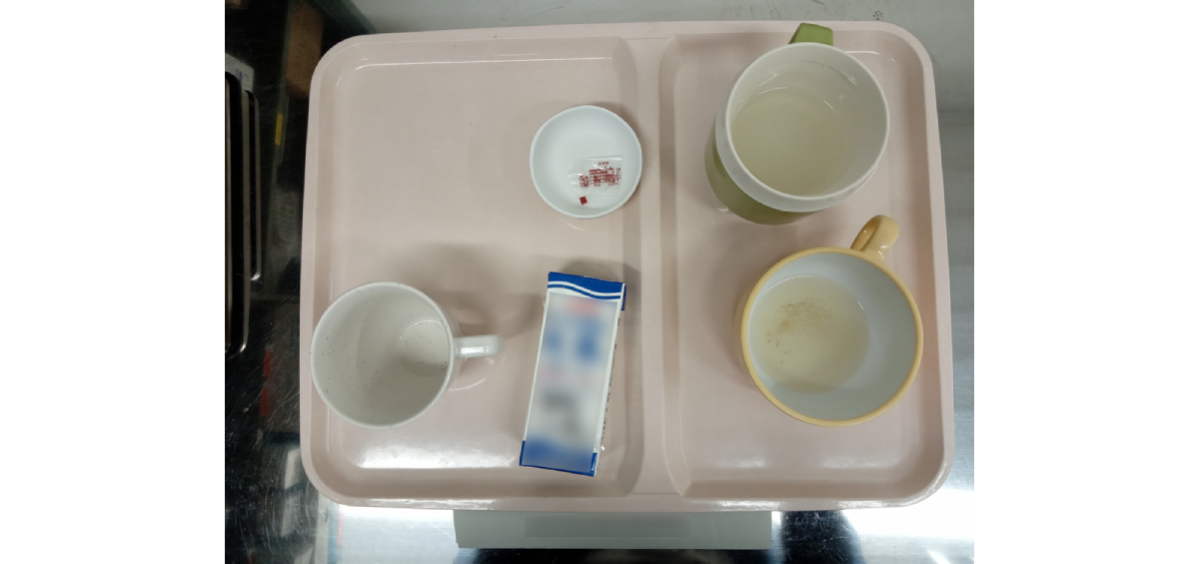
Example of liquid food image after the meal of a patient measured.

A total of 100 liquid foods provided to patients in 2 wards of Tokushima University Hospital between November 2020 and March 2021 were measured. For each dish, three types of measurements were performed: (1) image analysis evaluation of the food photo by an AI model through a picture after the meal (AI estimation), (2) visual measurement evaluation by a dietitian who looked at the same photo (image visual estimation), and (3) visual measurement evaluation by a nurse who looked directly at the food (direct visual estimation), and measurement by weighing. Figure 3 shows an image of a patient’s liquid food after their meal that was measured. The liquid food menu comprised a combination of staple food, side dishes 1, side dishes 2, packaged beverages, and seasonings. The lunch menus served during the study period were measured: staple food, thin rice gruel; side dish 1, vegetable soup; and side dish 2, fermented milk or peach, grape, orange, or mixed juice. Table 2 presents the types of dishes and number of liquid foods measured, and Table 3 presents the nutrients. Packaged beverages and seasonings were excluded as it was difficult to measure them through visual estimation. The operation is designed so that meals are not provided in advance when leaving the hospital or going out.

**Table 2:** Types of dishes and number of liquid foods measured.

Type of food and liquid food name			Measuring images, n
Staple food: thin rice gruel			100
Side dish 1: vegetable soup			100
**Side dish 2**			
	Fermented milk	31	
	Peach juice	18	
	Grape juice	12	
	Orange juice	13	
	Mixed juice	26	

**Table 3:** Nutrients in liquid foods.

Liquid food name	Energy (kcal)	Protein (g)	Fat (g)	Carbohydrate (g)
Thin rice gruel	32	0.5	0.1	7.0
Vegetable soup	3	0.1	0	0.5
Fermented milk	42	0.3	0	10.5
Peach juice	47	0.1	0.1	13.0
Grape juice	47	0.1	0.1	13.0
Orange juice	46	0.1	0.1	12.6
Mixed juice	66	0.5	0	13.7

### Measurement of Food Intake

#### Estimation Methods

The estimation method was rated on an 11-point scale as nurses usually measured food intake on an 11-point scale (0%, 10%, 20%, 30%, 40%, 50%, 60%, 70%, 80%, 90%, and 100%).

The AI estimation used food images photographed by the researcher on a tablet device (NEC LAVIE Tab E 8FHD1 camera resolution: 4160×3120, 72 dpi) after the nurse measured the food by direct visual estimation. The food images were resized from 4160×3120 to 960×720 as image preprocessing for AI image analysis. The AI model analyzed the food images uploaded to the food intake estimation system to estimate leftover food. Furthermore, the leftover amount was displayed on an 11-point scale. The amount of total food minus leftovers was calculated as the patient’s food intake.

The image visual estimation was performed by a dietitian, who visually estimated the food images photographed by the researcher. Processes of visual estimation were selected based on the medical staff’s routine work. Dietitians did not evaluate food intake as part of their routine work, but their nutritional assessment was performed in a usual clinical setting. Food intake was measured on an 11-point scale using the same food images photographed in the AI estimation. The measurements were recorded by the dietitian, who was informed of the study’s purpose and instructed on how to complete the measurement form in advance.

Direct visual estimation was performed by a nurse, who visually estimated the trays after the meals. The nurse collected the tray and immediately measured it on an 11-point scale. The nurse was also informed of the study’s purpose and instructed on how to complete the form in advance. Nurses routinely estimate food intake during their work.

#### Weighing Method

Data obtained from the hospital food provider’s information were used as the premeal weight, and the amount of liquid food provided was predetermined. Weighing measurements were performed on a digital scale (TANITA, 1458; maximum capacity: 1000 g, minimum display: 1 g, precision: ± 2 g) and recorded on an entry form by 2 researchers after the nurses performed the direct visual estimation. Food intake was calculated as the difference in weight before and after meals. The actual intake measured once using the weighing method was converted to an 11-point scale (Table 4).

**Table 4:** The converted values of actual measurement of food intake.

Converted value	Food intake
0	Ingesting 5% or less of the entire amount.
1	Ingesting between 5% and 15% of the entire amount.
2	Ingesting between 15% and 25% of the entire amount.
3	Ingesting between 25% and 35% of the entire amount.
4	Ingesting between 35% and 45% of the entire amount.
5	Ingesting between 45% and 55% of the entire amount.
6	Ingesting between 55% and 65% of the entire amount.
7	Ingesting between 65% and 75% of the entire amount.
8	Ingesting between 75% and 85% of the entire amount.
9	Ingesting between 85% and 95% of the entire amount.
10	Ingesting 95% or more of the entire amount.

### Accuracy Evaluation

The actual values from the weighing method were compared with estimates from the AI, image visuals, and direct visual estimations, as well as errors and distributions from each method for the staple food thin rice gruel, side dish 1 vegetable soup, and side dish 2 fermented milk or peach, grape, orange, and mixed juice. Corresponding *t* tests and Spearman rank correlation coefficients were used to verify the accuracy of the measurements through each estimation method. Nutrient intakes calculated from each estimation and weighing method were compared. Nutrient intake was calculated by multiplying the amount of nutrients in the menu items provided by the converted value of food intake. Bland-Altman plots were used to examine the differences between the estimated and actual values. Furthermore, the limits of agreement were calculated as the mean difference SD 1.96. In addition, the root-mean-square error (RMSE) and coefficient of determination (*R*^2^) were used as evaluation indicators to validate the measurement errors from the AI, image visual, and direct visual estimations [17,18]. The RMSE was a useful metric to develop AI models in which large errors were undesirable as it was weighted against large errors by squaring and averaging them. A smaller value indicates a smaller error and higher accuracy. The RMSE was calculated as follows:



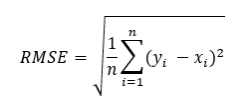



Here, *x* represents the estimated value (AI estimation, image visual estimation, direct visual estimation), and *y* represents the actual value (weighing method).

The *R*^2^ indicated the insignificance of the error compared with that of a model that always returned the average of the measured values. The closer this value is to 1, the higher its accuracy. *R*^2^ was calculated as follows:



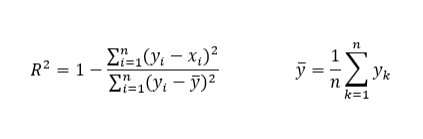



Mean error (ME) was used to determine whether the intake was overestimated or underestimated for each dish. ME was calculated as follows:



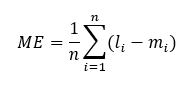



Here, m represents the converted estimated value of food intake, and l represents the converted actual value of food intake. The Friedman test was used to compare the mean differences in converted values of actual measurement of food intake by dish and estimation method to examine the errors between estimation methods that differed by dish. Finally, the MAE and a confusion matrix table of the estimated and actual values were created to evaluate the distribution of the absolute errors in the converted values for the actual measurement of food intake. It analyzed which intake of errors would be higher or the number of errors would be more frequent. The MAE was calculated as follows:



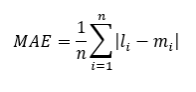



All statistical analyses were performed using SPSS Statistics (version 24; IBM Corp). *P*<.05 was deemed significant.

### Ethical Considerations

This study was approved by the Clinical Research Ethics Committee of Tokushima University Hospital (#3758).

## Results

Image visual estimations were performed by 6 dietitians from Tokushima University Hospital. Direct visual estimations were performed by 9 nurses from the University of Tokushima Hospital. Thin rice gruel as a staple food, vegetable soup, and fermented milk or peach, grape, orange, or mixed juices were evaluated. Table 5 presents the number of dishes by converted values of actual measurement of food intake. Table 6 presents summarized results regarding energy intake. There was no difference between the AI estimation and actual values. Spearman rank correlation coefficients were high with (ρ=0.89-0.97, *P*<.001). The AI estimation RMSE was smaller than the RMSE for image visual estimations, and the AI estimation *R*^2^ was higher than the *R*^2^ for image visual estimations.

**Table 5:** Number of dishes (N=300) by converted values of actual measurement of food intake (average food intake rate=84.7%).

Converted values of actual measurement of food intake	Number of dishes, n （%）
0	23 （7.7）
1	6 （2）
2	3 （1）
3	2 （0.7）
4	9 （3）
5	6 （2）
6	6 （2）
7	6 （2）
8	3 （1）
9	6 （2）
10	230 （76.7）

**Table 6:** The key results for energy intake.

	AI^a^ estimation	Image visual estimation	Direct visual estimation
Mean (SD) kcal	71.7 (23.9)	75.5 (23.2)	73.1 (26.4)
*P* value^b^	.82	<.001	.007
ρ^c^	0.89	0.94	0.97
RMSE^d^	8.12	8.49	4.34
R^2^	0.88	0.87	0.97

**^a^**AI: artificial intelligence.

^b^Difference between the mean of each estimated and actual measured value using a paired *t* test.

^c^Spearman rank correlation coefficient.

^d^RMSE: root-mean-square error.

### Differences Between the Estimated and Actual Measured Values

**Figure 4:**
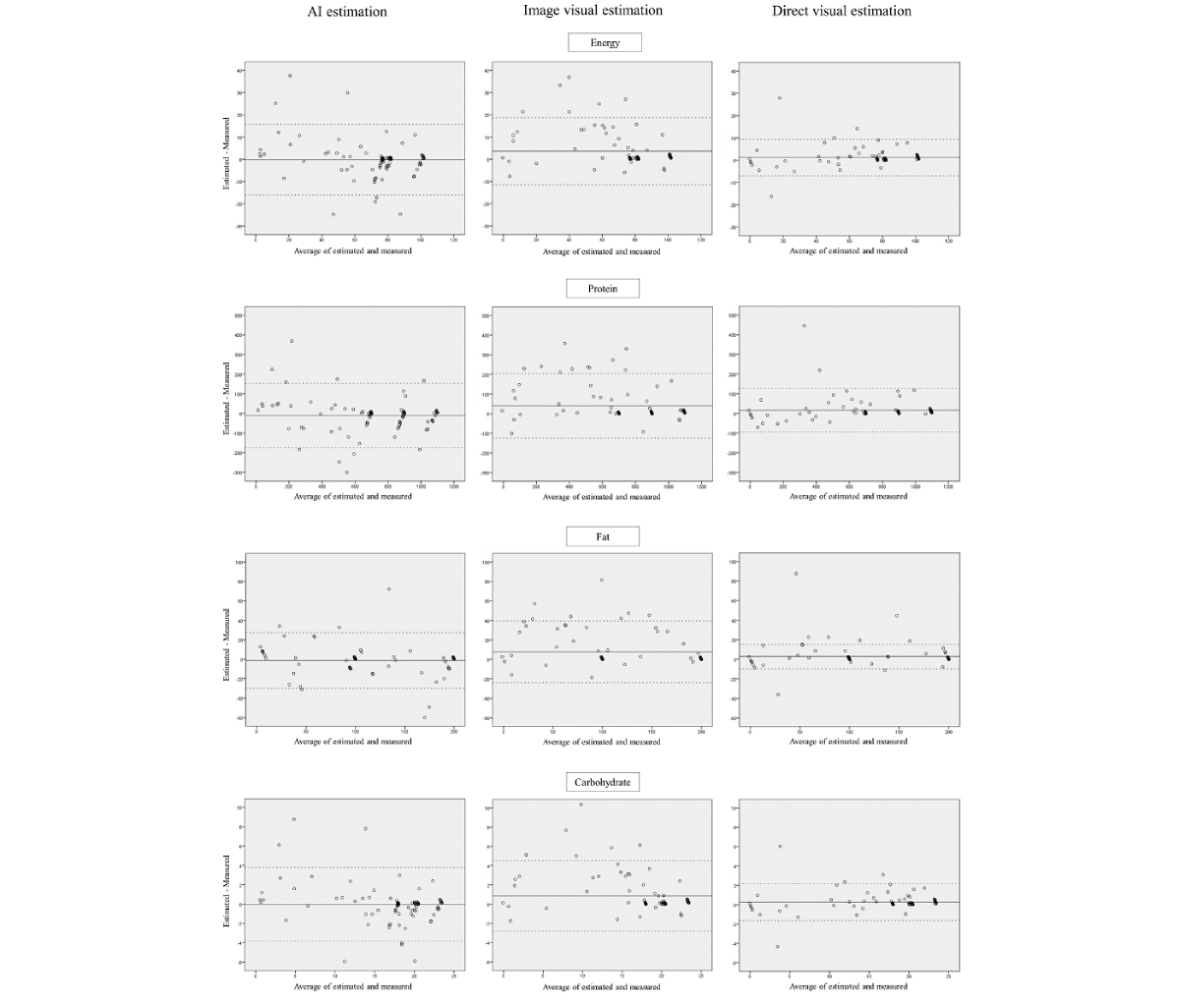
Bland-Altman analysis of the differences between each estimated and measured value of nutrient intakes.

Table 7 presents the estimated and actual measured mean nutrient intakes by the measurement method and their correlations. The mean nutrient intakes from the image and direct visual estimations were significantly different from those of the actual values. However, there was no difference between the AI estimation and actual values. Spearman rank correlation coefficients were high for all energy, protein, fat, and carbohydrate (ρ=0.89-0.97, *P*<.001). Correlation values for the AI estimation method were high (ρ=0.89-0.94); however, the direct visual estimation method generally showed higher correlations. Regarding the limits of agreement from the Bland-Altman plot, energy was AI estimation (–16.1 to 15.7 kcal), image visual estimation (–11.5 to 18.7 kcal), and direct visual estimation (–7.1 to 9.4 kcal); protein was AI estimation (–174.9 to 153.7 mg), image visual estimation (–124.0 to 203.2 mg), and direct visual estimation (–93.7 to 126.7 mg); fat content was AI estimation (–29.8 to 27.4 mg), image visual estimation (–24.2 to 39.4 mg), and direct visual estimation (–20.0 to 25.6 mg); and carbohydrate content was AI estimation (–3.8 to 3.8 g), image visual estimation (–2.8 to 4.5 g), and direct visual estimation (–1.6 to 2.2 g; Figure 4). All estimated values were highly correlated with the measured values. However, the limits of agreement for the Bland-Altman plots were wider for the AI and image visual estimation than for the direct visual estimation, and the agreement with the measured values was lower.

**Table 7:** Mean nutrient intake and Spearman rank correlation coefficient (ρ) between each estimated and actual measured value of nutrient intake.

		Measured value	Estimated value		
			AI^a^ estimation	Image visual estimation	Direct visual estimation
**Energy (kcal)**					
	Mean (SD) (kcal)	71.9 (26.2)	71.7 (23.9)	75.5 (23.2)	73.1 (26.4)
	*P* value^b^	—^c^	.82	<.001	.007
	ρ^d^	—	0.89	0.94	0.97
	*P* value^e^	—	<.001	<.001	<.001
**Protein (mg)**					
	Mean (SD) (mg)	723.2 (302.3)	712.6 (288.7)	762.8 (274.6)	739.7 (300.9)
	*P* value^b^	—	.21	<.001	.004
	ρ^c^	—	0.94	0.94	0.97
	*P* value^d^	—	<.001	<.001	<.001
**Fat (mg)**					
	Mean (SD) (mg)	116.1 (59.5)	114.9 (56.7)	123.7 (55.6)	118.9 (59.1)
	*P* value^b^	—	.41	<.001	.019
	ρ^c^	—	0.91	0.92	0.94
	*P* value^d^	—	<.001	<.001	<.001
**Carbohydrate (g)**					
	Mean (SD) (g)	17.2 (6.1)	17.2 (5.5)	18.0 (5.4)	17.5 (6.2)
	*P* value^b^	—	.93	<.001	.01
	ρ^c^	—	0.89	0.96	0.97
	*P* value^d^	—	<.001	<.001	<.001

^a^AI: artificial intelligence

^b^Difference between the mean of each estimated and actual measured value using a paired *t* test.

^c^Not applicable.

^d^Spearman rank correlation coefficient.

^e^Significance of correlation coefficients between each estimated and measured value using Spearman rank correlation coefficient.

### Differences Between Estimated Error Values

The RMSE for the AI estimation tended to be smaller and larger than those for image visual and direct visual estimations, respectively (Table 8). The coefficient of determination *R*^2^ for the AI estimation tended to be larger and smaller than those for image visual and direct visual estimations, respectively. In particular, the accuracy of orange juice by image visual estimation showed lower values (Table 9). The ME in the converted values of actual measurement of food intake by dish and measurement method were significant for the AI estimation and image visual estimation for staple thin rice gruel and side dish 1: vegetable soup. However, it was not significant for the other ME (Table 10).

**Table 8:** Differences between the actual measured values by nutrient and measurement method.

	AI^a^ estimation		Image visual estimation		Direct visual estimation	
	RMSE^b^	*R* ^2^	RMSE	*R* ^2^	RMSE	*R* ^2^
Energy	8.12	0.88	8.49	0.87	4.34	0.97
Protein	84.5	0.91	92.4	0.89	58.6	0.96
Fat	14.6	0.93	17.9	0.90	12.0	0.96
Carbohydrate	1.95	0.88	2.06	0.86	1.00	0.97

^a^AI: artificial intelligence.

^b^RMSE: root-mean-squared error.

**Table 9:** Differences between the actual measured values by nutrient, dish, and measurement method.

		AI^a^ estimation			Image visual estimation			Direct visual estimation	
		RMSE^b^	*R* ^2^	RMSE		*R* ^2^	RMSE		*R* ^2^
**Energy**									
	Thin rice gruel	3.96	0.99	4.68		0.99	3.61		0.99
	Vegetable soup	0.44	1.00	0.30		1.00	0.11		1.00
	Orange juice	2.85	0.99	11.3		0.86	0.60		1.00
	Fermented milk	5.94	0.97	3.18		0.99	1.38		1.00
	Grape juice	3.64	0.99	5.00		0.98	4.38		0.99
	Peach juice	5.45	0.97	2.75		0.99	1.38		1.00
	Mixed juice	8.10	0.85	5.89		0.93	1.72		1.00
**Protein**									
	Thin rice gruel	61.9	0.97	73.2		0.96	56.5		0.98
	Vegetable soup	14.7	0.81	9.91		0.91	3.60		0.99
	Orange juice	6.20	0.97	24.7		0.32	1.31		1.00
	Fermented milk	42.4	0.96	22.7		0.99	9.83		1.00
	Grape juice	7.75	0.96	10.6		0.91	9.32		0.95
	Peach juice	11.6	0.85	5.86		0.96	2.94		0.99
	Mixed juice	60.4	0.98	44.0		0.99	12.8		1.00
**Fat**									
	Thin rice gruel	12.4	0.86	14.6		0.77	11.3		0.89
	Vegetable soup	0	1.00	0		1.00	0		1.00
	Orange juice	6.20	0.97	24.7		0.32	1.31		1.00
	Fermented milk	0	1.00	0		1.00	0		1.00
	Grape juice	7.75	0.96	10.6		0.91	9.32		0.95
	Peach juice	11.6	0.85	5.86		0.96	2.94		0.99
	Mixed juice	0	1.00	0		1.00	0		1.00
**Carbohydrate**									
	Thin rice gruel	0.87	1.00	1.02		1.00	0.79		1.00
	Vegetable soup	0.07	1.00	0.05		1.00	0.02		1.00
	Orange juice	0.78	1.00	3.11		1.00	0.17		1.00
	Fermented milk	1.48	1.00	0.80		1.00	0.34		1.00
	Grape juice	1.00	1.00	1.37		1.00	1.20		1.00
	Peach juice	1.50	1.00	0.76		1.00	0.38		1.00
	Mixed juice	1.93	1.00	1.41		1.00	0.41		1.00

^a^AI: artificial intelligence.

^b^RMSE: root-mean-squared error.

**Table 10:** Mean errors in converted values of actual measurement of food intake by dish and measurement method.

Type of food	Liquid food name	Number of dishes	AI^a^ estimation	Image visual estimation	Direct visual estimation
Staple food	Thin rice gruel	100	–0.22^b^	0.54^b^	0.24
Side dishes 1	Vegetable soup	100	–0.53^b^	0.13^b^	–0.07
Side dishes 2	Orange juice	13	–0.15	0.85	0
Side dishes 2	Fermented milk	31	–0.06	0.19	0
Side dishes 2	Grape juice	12	0.25	0.50	–0.25
Side dishes 2	Peach juice	18	0.22	0	0.11
Side dishes 2	Mixed juice	26	0.08	0.23	0.04

^a^AI: artificial intelligence.

^b^Significant differences among the three measurement methods using the Friedman test (*P*<.05).

### Distribution of Errors Through the Confusion Matrix Table

**Figure 5:**
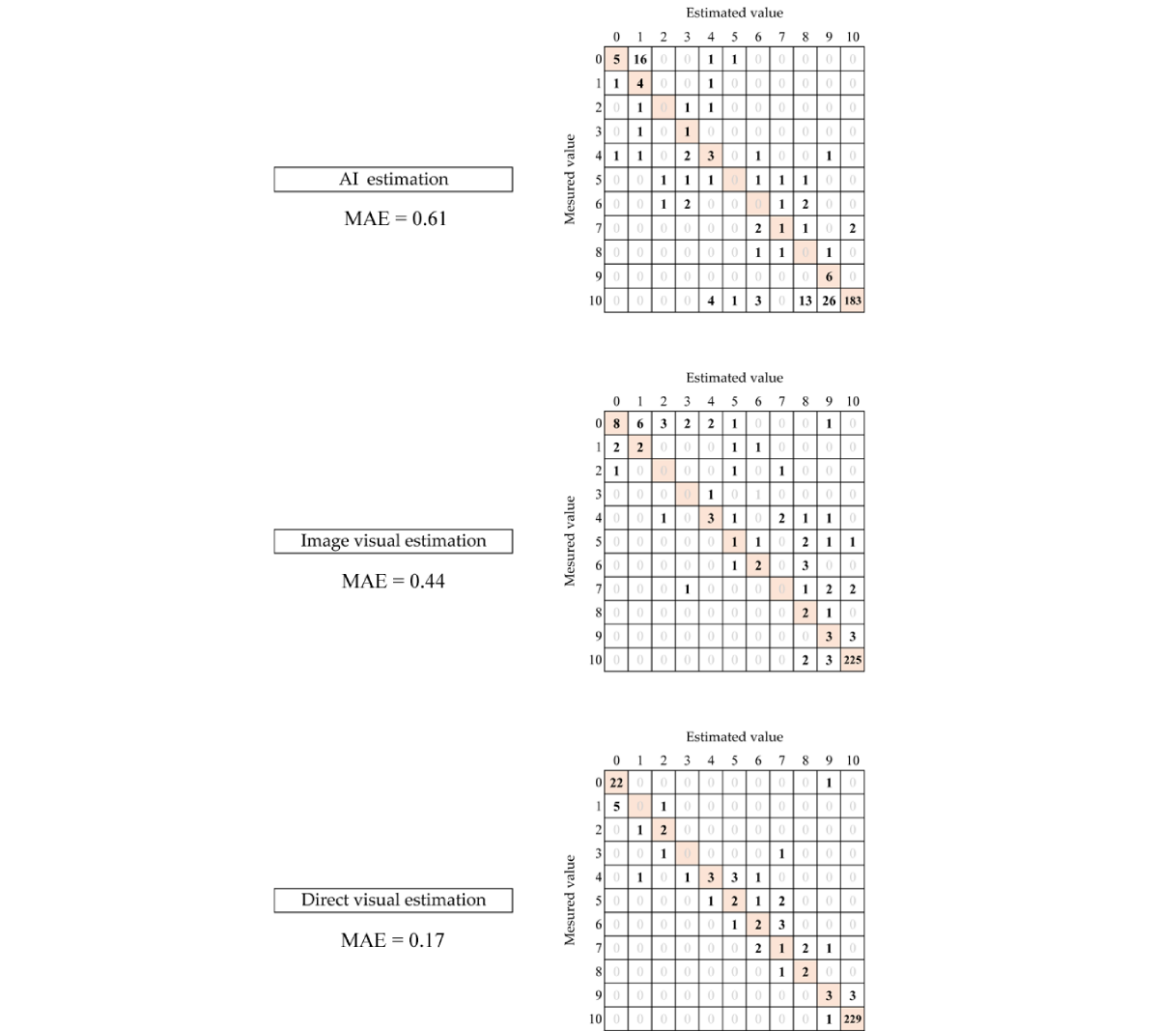
Confusion matrices of the measured and estimated values. AI: artificial intelligence. MAE: mean absolute error.

In the confusion matrix table of measured and converted values of the actual measurement of estimated food intake, the AI estimation showed more variation in the distribution of errors when the actual measurement was 10. Furthermore, the numbers of errors were larger (Figure 5). The image visual estimation also showed overall variation, and many evaluations estimated a higher intake than the measured values. Conversely, in direct visual estimations, the errors were small and almost consistent with the measured values. When the actual measured value was 0, there were a few errors. However, there was considerable variation in the distribution of errors in the AI and image visual estimations.

## Discussion

### Principal Findings

We examined the accuracy of food intake estimation through an original system that used an AI-based model to estimate leftovers for actual liquid food in a clinical setting. Our study showed that each estimation method was highly correlated with the weighing method based on the results that compared the respective estimates of the AI estimation by the food intake estimation system, image visual estimation by dietitians, and direct visual estimation by nurses by the weighing method. Furthermore, the AI estimation showed higher accuracy as there was no difference between the mean of the AI estimated and measured values. The AI estimation had an MAE of 0.61, which, when converted on an 11-point scale, had an error rate of 6.1%. This is more accurate than the 13.8% error reported in a previous study [19]. Furthermore, the AI estimation tended to have a smaller RMSE and larger coefficient of determination (*R*^2^) than image visual estimation. However, it tended to have a larger RMSE and smaller *R*^2^ than direct visual estimation, which resulted in lesser accuracy. Furthermore, the limits of agreement from the Bland-Altman plot were also wider.

This is the first study to compare an AI-estimated evaluation of liquid food consumed by patients with an evaluation estimated by medical staff based on actual food and food images. A previous study on AI systems has shown that several obstacles occur when transitioning from a research and development environment to practical use in a clinical context [20]. The AI estimation accuracy can have different levels of precision as it depends heavily on the training data. If the data have the same distribution as the training data, the AI model can make correct decisions. However, errors in practical situations can occur when data different from the training data are input [21]. Therefore, AI systems were significantly less accurate than reported prediction accuracy in a clinical context [22].

The AI and dietitians measured food intake through digital images captured from a mobile device. With the development of mobile technology, digital images are increasingly being used in food intake assessments [23]. Image visual estimation was reliable as human visual estimation of digital food images by a camera was highly correlated with the actual values measured by the weighing method [4,24]. Similarly, in this study, the estimates by the AI and image visual estimations were highly reliable as there was a high correlation with the values measured by the weighing method.

Various criteria have been used to evaluate the performance of AI models, including accuracy, computational speed, and interpretability. To evaluate the accuracy, this study converted the output to a continuous scale by averaging the classification results of multiple classification models and compared the error of each measurement, including the AI estimation, using the actual value measured by the weighing method as the correct answer. In this study, RMSE and *R*^2^ were used to evaluate the results. For the estimation error indicator of the continuous scale, RMSE and *R*^2^ were recommended when the prediction performance of the same scale was evaluated and when outliers were included, respectively [25]. Differences in the estimation error by type of food and intake were compared by the mean differences in the converted values of food intake and distributions in the confusion matrix table.

The results showed that the AI estimation had larger errors than the direct visual estimation. The reason was that the AI estimation was approximately 20% (47/230), which is in agreement with the measured value for the converted values of food intake 10, which was more than 95% of the total intake. Even when all the food was eaten, some food adhered to the inner sides and bottom of the dish, which could be mistakenly recognized as leftovers (eg, vegetables separated from the clear liquid in vegetable soup). Conversely, the direct visual estimation was in close agreement (Figure 5). Similarly, the results of the direct visual estimation in this study and previous studies reported that the correct rate for total intake was as high as 93%-96.4% [26-28]. Differences in the answers were related to the accuracy, as the average food intake rate in this study was 84.7%. Furthermore, 76.7% (230/300) of the evaluated dishes were classified as converted values of food intake 10. Results for the mean dietary intake rate were consistent with those of previous studies [29]. This suggested that differences in the percentage of correct answers for the converted values of food intake 10 that accounted for most of the dietary intake rate were related to the accuracy of the estimation in medical institutions.

In addition, image visual estimation had larger errors than direct visual estimation. A reason was that the visual image estimation showed a high degree of variability in the distribution of errors for the converted values of food intake 0, which was less than 5% of the total intake (Figure 5). The estimation for the converted values of food intake 0 was considered a difficult problem as there was considerable variation in the distribution of errors in the AI estimation; however, the direct visual estimation was almost consistent with the measured values. Therefore, difficulty in estimation could have been reduced by adding other information to determine intake, such as features not captured in the photographs that could indicate that the food was not touched when looking at it directly.

The accuracy of intake estimation could differ based on the type of dish and estimation method, as there was a difference in the error between AI and image visual estimations for thin rice gruel and vegetable soup (Table 10). The AI estimation tended to underestimate intake and evaluated it as smaller than the measured value. Conversely, image visual estimation tended to overestimate intake and evaluate it as larger than the measured value. Overestimation could have occurred as the evaluation was performed by looking at digital images. Previous studies reported that dietitians overestimated when they used visual estimation methods [29].

### Limitations

This study has several limitations. First, the photographed food images were used for the AI estimation. A previous study reported that direct visual estimation of actual food had a higher correlation with the weighing method than the visual estimation of food images [24]. In this study, direct visual estimation was more accurate than AI and image visual estimations. In addition, tablet-based food photography can be biased by differences in distance and angle. Packaged beverages were excluded from this study because it is difficult to evaluate leftover liquid foods with food images. For such foods, it is necessary to consider methods such as weighing.

Second, there was bias in the classification of the number of evaluations, as 76.7% (230/300) of the evaluated dishes were classified as converted values of food intake 10. Therefore, the number of dishes evaluated may not have been sufficient to evaluate the converted values as the number of dishes other than those converted to values of food intake 10 was small. The number of cases and types of dishes might be increased by adding breakfast and dinner, as only lunch was covered in this study.

Third, this evaluation was conducted at a single facility. The menus and tableware for hospital liquid food served to patients varied between facilities. The estimation accuracy and number of study images per facility should be investigated. In addition, solid food intake should be measured to evaluate multiple facilities and varied menus. Solid food does not have a constant, remaining food area, unlike liquid food. The volume of the remaining food area must be calculated to measure the amount of solid food remaining. Accurate measurement of food volume has been attempted by using equipment that allows for a constant angle of view and distance from the food image [19]. Therefore, it is necessary to consider the development of an AI model for solid food using such a device as the next step.

Finally, the usability of the proposed AI-based measurement method for medical staff remains unclear. Regardless of the system’s high accuracy, if its usability was low, it would be an obstacle during transitioning the system to practical use in clinical contexts [20]. For routine nutrition management, a system that uses AI image analysis to support meal recording has been evaluated for its simplicity and other usefulness [30-33]. AI-based measurements require usability assessments among medical staff in clinical environments. We are currently developing an automated meal tray photography device to improve usability for the measurer, and we are also planning a usability evaluation of an AI-based system for estimating food intake using this device.

### Conclusions

The measurement from the food intake estimation system through an AI-based model to estimate the leftover liquid food intake for patients showed a high correlation with the actual values by the weighing method and higher accuracy than the image visual estimation. However, the AI estimation was less accurate than direct visual estimation. Therefore, its accuracy could be further improved. We aim to improve AI accuracy by learning from significantly different estimation data. However, the errors of the AI estimation method were within the acceptable range of the weighing method, which indicated that the AI-based food intake estimation system could be applied in clinical settings. Accurate nutritional data is difficult to obtain because of the heavy burden on medical staff. AI-based measurements are expected to reduce this burden and improve nutritional management.

## Abbreviations

AI: artificial intelligence
MAE: mean absolute error
ME: mean error
RMSE: root-mean-squared error
